# An Open-Source R Package for Detection of Adverse Events Under-Reporting in Clinical Trials: Implementation and Validation by the IMPALA (Inter coMPany quALity Analytics) Consortium

**DOI:** 10.1007/s43441-024-00631-8

**Published:** 2024-04-02

**Authors:** Björn Koneswarakantha, Ronojit Adyanthaya, Jennifer Emerson, Frederik Collin, Annett Keller, Michaela Mattheus, Ioannis Spyroglou, Sandra Donevska, Timothé Ménard, On behalf of the IMPALA (Inter coMPany quALity Analytics) Consortium

**Affiliations:** 1 F. Hoffmann-La Roche AG, 4070 Basel, Switzerland; 2 Merck & Co., Inc., Rahway, NJ 07065 USA; 3 Boehringer Ingelheim Pharma GmbH & Co, 88397 Biberach an der Riss, Germany; 4 Boehringer Ingelheim Pharma GmbH & Co, 55216 Ingelheim, Germany

**Keywords:** Quality assurance, Open-source, Analytics, GCP, Validation, Transparency

## Abstract

Accurate and timely reporting of adverse events (AEs) in clinical trials is crucial to ensuring data integrity and patient safety. However, AE under-reporting remains a challenge, often highlighted in Good Clinical Practice (GCP) audits and inspections. Traditional detection methods, such as on-site investigator audits via manual source data verification (SDV), have limitations. Addressing this, the open-source R package {simaerep} was developed to facilitate rapid, comprehensive, and near-real-time detection of AE under-reporting at each clinical trial site. This package leverages patient-level AE and visit data for its analyses. To validate its efficacy, three member companies from the Inter coMPany quALity Analytics (IMPALA) consortium independently assessed the package. Results showed that {simaerep} consistently and effectively identified AE under-reporting across all three companies, particularly when there were significant differences in AE rates between compliant and non-compliant sites. Furthermore, {simaerep}'s detection rates surpassed heuristic methods, and it identified 50% of all detectable sites as early as 25% into the designated study duration. The open-source package can be embedded into audits to enable fast, holistic, and repeatable quality oversight of clinical trials.

**Supplementary Information:**

The online version contains supplementary material available at 10.1007/s43441-024-00631-8.

## Background

### Adverse Events in Quality Assurance of Clinical Trials

The quality of clinical trial data is crucial to ensure patient safety and compliance to Good Clinical Practices, GCP [1, 2]. Adverse events (AEs) are collected during clinical trials to assess the drug's safety profile, and therefore it is critical that AEs are reported timely to the sponsor. Detecting quality risks related to AE under-reporting is a key component of quality assurance (QA) oversight of clinical trials [1–3]. Inadequate reporting of adverse events (AEs) from investigator sites is one of the GCP inspection findings [4–6]. In some cases, AE under-reporting has caused delays in regulatory submissions or the non-approval of new drugs [6, 7]. Current clinical QA practices heavily rely on on-site audits to identify issues, including AE under-reporting. However, the increasing number of trials and sites, as well as the complexity of study designs, makes it difficult to detect AE under-reporting. Current site monitoring strategies, such as on-site source data verification (SDV) and risk-based approaches, attempt to address this issue [8]. Nevertheless, AE under-reporting remains a common finding during audits and inspections [4, 5]. Over the last few years, the industry has been exploring modern developments in data management and IT systems to facilitate cross-analysis of clinical studies [9, 10]. In this analytical report, we aim to focus on the validation and reproducibility of an open-source analytics package that could be used across the ecosystem to tackle the issue of AE under-reporting.

### Bootstrap Resampling Algorithm

The bootstrap resampling open-source R package {simaerep} was initially developed and deployed within one of the member companies [11, 12]. The open-source code was publicly shared and reviewed by IMPALA members. Based on the reviews, new features were collaboratively added and version 0.4.3 is currently available on CRAN [13]. Its algorithm employs a resampling methodology to predict AE under-reporting at clinical trial sites. The method assumes that all patients follow the same visit schedule and that the probability for having an AE at a specific visit is the same for all patients. It reshuffles patients between sites of one study and then experimentally determines how often any observed AE reporting behavior randomly occurs. It requires only AE and subject visit data of the study of interest and does not need any specific AE, site, or subject characteristics apart from the cumulative AE and visit counts on the study and site levels. As patients join the trial at different calendar times, not all of them will be under observation for the same duration. To include as many patients covering the same reasonably high visit count, we determine a site-specific evaluation point by determining the median of all maximum consecutive visit counts of all patients multiplied by 0.75 (visit_med75). All patients that have not reached the visit_med75 will be excluded from the evaluation. While patients are progressing in the study, the visit_med75 will increase and finally 100% of all patients completing the study will be included. Then the average number of AEs per patient at the visit_med75 at site is calculated and it is then measured how often we can experimentally obtain a value that is equal or lower if we reassign random patients from the study pool to that site. To control for multiplicity, the resulting probabilities can be corrected using the Benjamini–Hochberg method [14].

This method is non-parametric and does not require any assumptions about the statistical distribution of occurrences of adverse events. But it makes the strong assumption that all sites have access to the same patient pool (of all patients in the study), which is not necessarily realistic as it has already been demonstrated that sites from central and eastern Europe may report on average a lower number of AEs [11].

### IMPALA: Cross-Industry Implementation

The IMPALA (Inter coMPany quALity Analytics) group was formed in July 2019 and launched as a non-profit consortium in October 2022 [15, 16]. One of its goals is to address the limitations of traditional QA approaches by co-developing advanced analytics and best practices that can help detect and mitigate issues faster, reduce the burden of retrospective, time-consuming traditional QA activities, and ultimately accelerate approval and patient access to innovative drugs [15, 16].

Three pharmaceutical sponsor companies which are members of IMPALA (Roche, Merck Sharp & Dohme LLC, a subsidiary of Merck & Co., Inc., Rahway, NJ, USA, Boehringer Ingelheim) tested and then implemented the {simaerep} open-source package in order to evaluate its suitability for broader adoption, i.e., by multiple sponsors but also by health authorities’ inspection teams.

## Methods

IMPALA members had been tasked to independently evaluate the usage of the {simaerep} algorithm for the detection of AE under-reporting sites.

The evaluation exercise provided several challenges that each company resolved differently in their approach:

- *Evaluation Data Set* there was no standardized test data set for AE under-reporting, and therefore a realistic study data set or simulation thereof needed to be selected.
- *Evaluation Endpoint* it was not defined which quantity of unreported AEs qualified as AE under-reporting. The {simaerep} algorithm assumes that under-reporting is quantitatively distinguishable from compliant reporting. These quantitative under-reporting scenarios needed to be clearly defined by the study under-reporting ratio (Study-UR) which we define as the ratio of sites within a study that under-report AEs; and the site under-reporting ratio (Site-UR) which defines the ratio of unreported AEs at a site. Additionally, a classification cut-off for the under-reporting probability as returned by the {simaerep} package needed to be determined.
- *Site Heterogeneity* AEs are reported by several sites which are often spread around the globe each with access to a unique patient population with different cultural traits of AE reporting [17]. The {simaerep} algorithm does not account for site heterogeneity.
- *Study Heterogeneity* AE rates and volume are highly dependent on the study protocol and design (including studied population, therapeutic indication, study and comparator drug). The more AEs have been reported, the easier it is to distinguish between compliant and non-compliant low AE rates.
- *Time Dimension* in practice, it is necessary to detect under-reporting as early as possible during a trial. Evaluation should include ongoing trial data.

The evaluation strategies are summarized in Table 1 and details can be found in the Supplementary Materials.

**Table 1:** Methodology of {simaerep} Validation Approaches.

	Roche	Boehringer Ingelheim	Merck
Data Set	Simulated data set based on current study portfolio snapshot measuring site patient and visit counts and study AE rates	Simulated data set consisting of one large- and one medium-size trial	Historic studies with AE-related protocol deviations
Evaluation Endpoint	Detection of single under-reporting sites with varying Site-URs: 0.1, 0.25, 0.5, 0.75, 1	Detection of under-reporting sites in two scenarios Large scenario: Study-UR: 0.1633 Site-UR: 0.25–0.75 Medium Scenario: Study-UR: 0.0523 Site-UR: 0.25	Detection of sites with AE-related protocol deviations
Classification Cut-off	95%	The cut-off was determined employing the Youden method for each experimental condition maximizing TPR and FPR	50%
Time Dimension	Snapshot includes early-, mid-, and late-stage trials	The AE reporting was simulated over time	Assessment on the day an AE-related protocol deviation had occurred
Site Heterogeneity	Sites differed by number of visits and patients	Sites differed by number of patients, date of entry into the study (different across regions), and AE reporting rate (different across regions)	Realistic site heterogeneity that can be found in clinical trial data sets. Sites might differ by number of patients and visits, date of study entry, regional AE rates, and access to unique patient populations
Study Heterogeneity	Portfolio-based trial heterogeneity Studies with less than 100 patients and less than 10 sites were excluded	Two sizes of trial: large (7000 patients over 490 sites) and medium (1545 patients over 172 sites) size scenarios	Portfolio-based trial heterogeneity Studies with less than 100 patients and less than 10 sites were excluded
Uniqueness of the Approach	Compares performance to standard heuristic under-reporting methods based on boxplot outliers and rank of AE rates	Blinded experimental approach separating data simulation and data evaluation Aimed to detect the earliest time point at which under-reporting appears	Does not rely on simulations but uses real data
{simaerep} Parameter Settings	Adjusted vs. unadjusted visit_med75, AEs per day vs. AEs per visit	With multiplicity correction and without	Default (with multiplicity correction)

Performance was evaluated using the true positive rate (TPR) which measures the proportion of actual under-reporting sites that are correctly identified. A higher TPR is desirable, with the ideal value being 1, indicating perfect identification of all positive cases. The false positive rate (FPR) quantifies the proportion of sites with compliant AE reporting that are falsely flagged as under-reporting. The FPR should be as close to 0 as possible, indicating no incorrectly flagged sites.

The Roche approach measured {simaerep} performance across a simulated data set based on a snapshot of an entire portfolio of ongoing studies. Each study was simulated to have only one under-reporting site for which under-reporting probabilities for Site-URs of 0.1, 0.25, 0.5, 0.75, 1 were calculated. This was iteratively repeated for each site in the study. A classification cut-off of 95% would determine whether it was flagged for under-reporting. TPR and FPR were also compared to standard heuristic methods.

The approach chosen by Boehringer Ingelheim was to select two simulated trials mirroring studies that could have occurred. Two data sets, one large trial with high AE reporting rates (Study-UR: 0.25, Site-URs 0.25–0.5) and a medium-sized trial with lower AE reporting rates (Study-UR 0.0523, Site-UR 0.25) were generated also accounting for differences in AE reporting rates across geographic regions. To define the classification cut-off, the Youden method [18], which maximizes TPR and FPR, was employed for each experimental condition.

Merck’s approach calculated AE under-reporting probabilities with {simaerep} for sites in historical trials with AE-related protocol deviations. Among those, a classification cut-off of 50% was chosen to calculate the TPR. AE under-reporting probability was reported for sites with AE-related protocol deviations calculated using a snapshot of the entire study data. AE under-reporting probabilities for sites without protocol deviations were not reported and hence no FPRs could be calculated.

**Figure 1:**
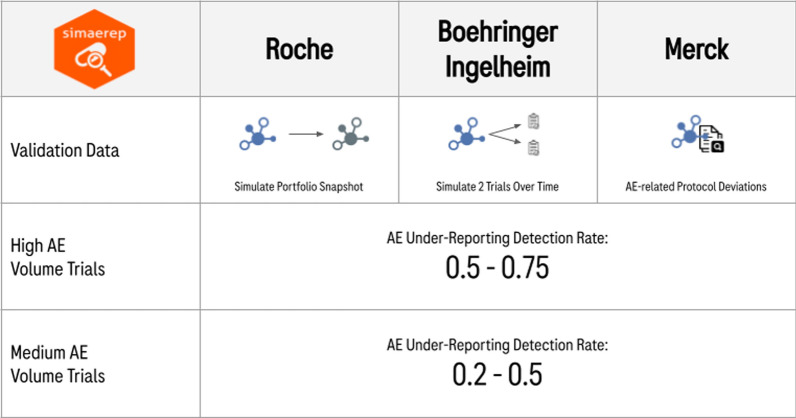
Different Validation Approaches Show Efficient Detection of AE Under-reporting Sites Using {simaerep}.

As these approaches are very different, the resulting performance metrics were difficult to compare. We therefore categorized the different scenarios tested by each approach by how challenging it was to detect AE under-reporting under these conditions. It is to be expected that the detection of AE under-reporting will be more successful the higher the quantitative difference is between AEs reported by compliant and non-compliant sites. The total volume of AEs depends on the three factors: (i) total number of patients, (ii) the number of visits or the time spent in the trial, and (iii) the time-adjusted AE rate. Based on the reported parameters, we classified each experiment either into a high AE volume scenario or a medium AE volume scenario (see Fig. 1; Table 2).

**Table 2:** Results of {simaerep} Validation Approaches for Scenarios with High or Average AE Volume.

	Roche	Boehringer Ingelheim	Merck
High AE Volume Scenario: Description	No. patients 200 No. sites 20 No. patients per site 10 No. under-reporting sites 1 AEs per visit 0.5 Visits per patient 20 (average) Site-UR: 0.25–0.75	No. patients 7000 No. sites 490 No. patients per site 2–34, median 13 No. under-reporting sites 80 AEs per subject and year 0.89 Site-UR: 0.25–0.75	Portfolio sites with visit_med75 ≥ 10
High AE Volume Scenario: Result	FPR: 0.002 Site-UR 0.1 with TPR: 0.028 Site-UR 0.25 with TPR: 0.272 Site-UR 0.5 with TPR: 0.976 Site-UR 0.75 with TPR: 1	FPR: 0.1488 TPR: 0.875	FPR: not calculated Patients ≥ 5 TPR: 0.459 Patients ≥ 10 TPR: 0.7142
High AE Volume Scenario: Combined performance estimate (All Companies)	FPR < 0.01 Site-UR 0.25–0.5 with TPR > 0.5 Site-UR 0.5–0.75 with TPR > 0.75	FPR < 0.01 Site-UR 0.25–0.5 with TPR > 0.5 Site-UR 0.5–0.75 with TPR > 0.75	FPR < 0.01 Site-UR 0.25–0.5 with TPR > 0.5 Site-UR 0.5–0.75 with TPR > 0.75
Medium AE Volume Scenario: Description	Portfolio	No. patients 1545 No. sites 172 No. patients per site 1–42, median 9 No. under-reporting sites 9 AEs per subject and year 0.58 Site-UR: 0.25	Portfolio
Medium AE Volume Scenario: Result	FPR: 0.014 Site-UR 0.1 with TPR: 0.062 Site-UR 0.25 with TPR: 0.213 Site-UR 0.5 with TPR: 0.493 Site-UR 0.75 with TPR: 0.695	FPR: 0.405 TPR: 0.889	FPR: not calculated TPR: 0.20
Medium AE Volume Scenario: Combined performance estimate (All Companies)	FPR < 0.025 Site-UR 0.25–0.5 with TPR > 0.2 Site-UR 0.5–0.75 with TPR > 0.5	FPR < 0.025 Site-UR 0.25–0.5 with TPR > 0.2 Site-UR 0.5–0.75 with TPR > 0.5	FPR < 0.025 Site-UR 0.25–0.5 with TPR > 0.2 Site-UR 0.5–0.75 with TPR > 0.5
Additional Observations	{simaerep} detection followed a similar pattern as detection using boxplot outlier statistics for AE rates with {simaerep} having more favorable TPR and FPR. Default settings for {simaerep} could be confirmed	Better performance was obtained without correcting for multiplicity. 50% of all correctly detectable under-reporting sites were detectable at 25–32% of study time passed after the first visit	30% of all cases with AE-related protocol deviations had above average AE rates Trials from therapeutic areas (TA) with high enrollment such as vaccine trials would have TPR rates of up to 0.39 compared to 0.2 (all TA)

## Results

The adequacy of the {simaerep} algorithm has been independently evaluated by three different companies. Two companies took a simulation-based approach in which they simulated study data based on real ongoing or completed studies with subsequent introduction of under-reporting. Another approach was to detect sites for which AE-related protocol deviations were recorded.

In order to compare the performance metrics of each approach, we have categorized the different scenarios into either a high volume AE scenario or a medium volume AE scenario. The main findings are summarized in Fig. 1. Under high AE volume conditions, the detection of under-reporting is easier and we can detect 50–75% of all sites with AE under-reporting. With a medium AE volume in a trial, we can still detect 20–50% of all under-reporting sites.

These results depend on various other scenario parameters that are summarized in Table 2 and more details can be found in the Supplementary Materials.

For the high AE volume scenario, the “optimal study conditions” scenario included in the evaluation of the Roche approach was picked. It was not based on any study in the portfolio but a scenario with fixed simulation parameters that emulates a study with high AE rates. From the Boehringer Ingelheim approach, the large simulated trial provides a similar scenario. Merck stratified their reported results by the number of patients on site and the selected evaluation visit point (visit_med75) which was determined by the median of the maximum visit count of each patient multiplied by 0.75. Both numbers of patients and visits directly correlate with the total AE count expected at that site. Hence, the Merck analysis including only sites with visit_med75 ≥ 10 was selected as the high AE volume scenario.

Maximum TPRs were 1.000 (Roche), 0.875 (Boehringer Ingelheim), and 0.714 (Merck). In the Roche scenario, with its many similar sites each hosting 10 patients and frequent visits, detecting under-reporting is relatively easy. However, in the scenarios from Boehringer Ingelheim and Merck, the sites vary more and also include sites with fewer patients and visits, which makes detecting under-reporting harder. Collectively, this showed that under conditions with high AE rates high TPRs can be obtained using the algorithm. Decreasing the number of patients in the Merck scenario (or the rate of under-reporting in the other scenarios) increased the detection difficulty and reduced the TPR. Roche’s ideal scenario reported a very low FPR of 0.002, while Boehringer Ingelheim’s more heterogeneous scenario reported a fairly high FPR of 0.1488. Depending on the subsequent action, the Boehringer Ingelheim TPR would need to be reduced in order to bring the FPR down to a more practical level. We can try to combine these empirical results into likely performance estimates for the future application of {simaerep}. These estimates are not universal as they are dependent on the volume of AEs, the tolerable FPR, and the rate at which non-compliant sites are under-reporting AEs. They represent estimated averages from all three results. Altogether, this suggests for high volume AE scenarios and a targeted FPR < 0.01 the results suggest a TPR > 0.5 (Site-UR 0.25–0.5) or a TPR > 0.75 (Site-UR 0.5–0.75).

For studies with medium AE volume, we compared the portfolio-based results of the Roche and the Merck approach with the medium trial scenario of the Boehringer Ingelheim approach. Merck reported a TPR of 0.2, Boehringer Ingelheim reported a TPR of 0.889 at the cost of a very high FPR of 0.405 for a fixed under-reporting rate of 0.25, and Roche reported TPR of 0.213, 0.493, and 0.695 for Site-URs of, respectively, 0.25, 0.5, and 0.75. We can combine these results into the following estimates as we did for the high AE volume scenarios. For medium AE volume scenarios, sites with a Site-UR of 0.25 can expect a TPR of approximately 0.25. Sites with higher Site-URs of 0.5–0.75 can expect a TPR of greater than 0.5. For these TPR rates for medium volume AE scenarios, the expected FPR rate is approximately 0.025. When higher FPR rates can be tolerated, higher TPR rates can be obtained (see Boehringer Ingelheim approach).

These statements expect that the Study-UR in a medium AE volume trial is low and does not exceed the highest ratio tested in the Boehringer Ingelheim experiments of 0.16.

Each evaluation approach included some features that generated unique insights. The Roche approach compared {simaerep} against heuristic detection methods and found that the {simaerep} flagging pattern followed a similar pattern as flagging by boxplot outlier statistics of site AE rates. However, flagging on the basis of {simaerep} under-reporting probability provided more favorable TPR and FPR. Moreover, it was found that the algorithm's default settings provided the best performance. Roche also tried to detect under-reporting for low levels of under-reporting (Site-UR 0.1) resulting in TPR lower than 0.07 which is too low to be relevant. This implies that Site-UR should be greater than 0.25 to effectively detect under-reporting. In the context of the Boehringer Ingelheim approach, it was found that the Benjamini–Hochberg [14] multiplicity correction offered by {simaerep} was too conservative and reduced overall classification performance expressed by the receiver operating characteristics area under the curve (ROC AUC), a metric independent of classification cut-off threshold [19]. Additionally, we noted that approximately 50% of all sites with accurately detectable under-reporting were identifiable as early as when 25% of the study time had elapsed following the first visit in the medium trial, and 32% in the large trial.

Merck has reported that performance varied between therapeutic areas and that therapeutic areas with high enrollment such as vaccine trials had the highest TPR rates. Merck also noted that in 30% of the sites with AE-related PD, the average AE reported by the site at visit_med75 was higher than the site-level average AE reported by the study. These instances cannot be detected by the algorithm because it only identifies sites with a (significantly) lower average than the study.

## Discussion

The {simaerep} R package uses a bootstrap-resampling-based algorithm to calculate AE under-reporting probability in order to detect AE under-reporting sites in clinical trials [11, 12]. The performance of the package has been independently evaluated by three different companies using different strategies. By the separation of the individually obtained results into scenarios with high and medium AE reporting rates, we could make combined estimates about expected performance metrics. We could also show that adequate detection of under-reporting sites can be already obtained after roughly 30% of the allocated study time has passed. When compared to more simple heuristic-based strategies, the results obtained by using {simaerep} allowed for more accurate detection of AE under-reporting. A limitation of the independent validation approach is that these ‘additional observations’ could not be verified by the approaches of the other companies. On the other hand, this flexible validation approach allowed for more ‘additional observations’ to be made in the first place. Furthermore, only the Boehringer Ingelheim team was able to resource independent teams to their validation approach to control for internal biases.

Various classification cut-off thresholds were tested. In general, it was demonstrated that lowering the cut-off for classification of under-reporting will increase the TPR but will increase the FPR at the same time. In general, it should be recommended to set the cut-off based on the desired FPR which depends on how the algorithmic results are connected to business processes. Usually quality activities are very costly and resource intensive [20] and it is advisable to lower the expected FPR rate as much as possible.

In clinical trials, AE under-reporting is currently either detected by on-site personnel and filed as a self-identified protocol deviation or it is detected during an audit or inspection [1, 4]. The root cause is usually that single AEs have not been entered from the patients’ source record into the clinical database. This often only affects a small number of AEs and hence does not change the quantitative volume of AEs reported by a site. Merck has shown that this was the case for 30% of all AE under-reporting-related protocol deviations in their portfolio. AE under-reporting issues that are detectable by {simaerep} need to affect the overall AE volume at a site and thus need to be systemic and reoccurring. The root cause for these issues can be numerous: patients not being questioned for AEs, AEs not entered into the source files, AEs not entered into the clinical database, cultural differences in AE reporting [17], patients clusters that are healthier than the study average.

All three evaluation strategies have addressed the potential heterogeneity of studies that {simaerep} was suitable for. In general, adequate detection rates could be obtained for all studies with medium to high AE rates. Studies with a low volume of AE reporting can nevertheless be analyzed and under-reporting can be detected. Should the cut-off for classification of under-reporting be lowered to accommodate for the weaker statistical signal, a higher FPR should be expected.

There are potential root causes that can affect the volume of AEs reported that can be categorized as compliant site behavior, for example, cultural differences in AE reporting or a cluster of healthier than average study participants. This creates some heterogeneity across sites that cannot be accounted for by {simaerep} algorithm. The Boehringer Ingelheim approach has taken this into account and addressed this in their simulations. The {simaerep} open-source analytics package can be integrated into the framework of an ‘analytics-based audit’ and represents a significant advancement in the ability to conduct rigorous audits of clinical trials, focusing on safety reporting and sponsor oversight [21]. It allows auditors to depart from traditional, limited sampling methods and instead scrutinize an entire program (of multiple clinical trials) comprehensively. By employing {simaerep}, audits may gain a substantial boost in efficiency and increase the probability of identifying potential issues or anomalies of safety reporting. This comprehensive approach may also enable fast, holistic, and repeatable quality oversight of clinical trials. This integration fundamentally transforms the audit process, enhancing its accuracy and effectiveness, and ultimately contributes to the goal of improving the safety and quality of clinical trials.

## Conclusion

Cross-industry collaboration is pivotal for developing new standards for quality analytics. Here, we demonstrated how IMPALA members could collaborate to evaluate and improve innovative QA analytics packages. IMPALA currently has other work products and packages that are being co-developed [16] and will continue to proactively and transparently share these to accelerate innovation for clinical QA.

Using {simaerep}, we could consistently flag sites with lower than expected AE reporting rates, which allows us to manage, target, and focus QA activities to minimize quality risks. This package enables early decision-making to maximize clinical trials’ quality, which can accelerate the development of new medicines and improve patient outcomes. Through open-source publishing and collaboration, we aim to set new standards for advanced analytical methods for QA while being transparent on how new methods and algorithms were developed and validated.

### Supplementary Information

Below is the link to the electronic supplementary material.

Supplementary file1 (PDF 577 KB)

## Data Availability

Data used for this publication are based on ongoing clinical trials and cannot openly be shared. Code for the Roche validation approach is part of the {simaerep} documentation including suggestions on how to simulate appropriate validation data based on real or manually specified study portfolios.

## Code Availability

The code is available at https://github.com/openpharma/simaerep/.
