# Supplementary material for: An Open-Source R Package for Detection of Adverse Events Under-Reporting in Clinical Trials: Implementation and Validation by the IMPALA (Inter coMPany quALity Analytics) Consortium
Source: Ther Innov Regul Sci. 2024 Apr 2;58(4):591–9. doi: 10.1007/s43441-024-00631-8 (PMC11169048; doi:10.1007/s43441-024-00631-8)
Supplement: Supplementary file 1 — Supplementary file1 (PDF 577 KB) [file 43441_2024_631_MOESM1_ESM.pdf]

An Open Source R Package for Detection of Adverse Events under-reporting in Clinical Trials - Implementation and Validation by the IMPALA (Inter coMPany quALity Analytics) Consortium - Supplementary Materials

Bjorn Koneswarakantha[1] • Ronojit Adyanthaya[2] • Jennifer Emerson[3] • Frederik Collin[3] • Annett Keller[3] • Michaela Mattheus[3] • Ioannis Spyroglou[2] • Sandra Donevska[2] • Timothé Ménard[1] - On behalf of the IMPALA (Inter coMPany quALity Analytics) consortium

1. F. Hoffmann-La Roche AG - CH-4070, Basel, Switzerland
   corresponding author: timothe.menard@roche.com
2. Merck & Co., Inc., Rahway, NJ 07065, United States of America
3. Boehringer Ingelheim Pharma GmbH & Co KG- 88397 Biberach an der Riss, Germany

Background for the Supplementary Materials

In these supplementary materials, we are providing further details on the methodology and results from the three sponsors (Roche, Boehringer-Ingelheim, Merck) which evaluated the performance of {simaerep}.

Methods

Roche approach

To assess the performance of the AE under-reporting detection tool, we employed a simulation-based approach. We generated artificial portfolio data that mirrors our real-world study portfolio of ongoing studies with consistent AE reporting rates across all sites in a given study. We would then test {simaerep} performance in different AE under-reporting scenarios (under-reporting ratios: 0.1, 0.25, 0.5, 0.75, 1).

To simulate the portfolio, we determined for every site the number of patients, the average number of total visits per patient, the standard deviation (SD) of the total number of visits per patient and for every study the average AE per visit rate. These parameters were used to sample the effective total number of patient visits from a normal distribution and the total AE count per visit from a Poisson distribution.

Any AE under-reporting detection method will be challenged in scenarios in which a low number of AEs are expected e.g. studies with healthy participants, studies that have just started. And many of these challenging scenarios can be found in our portfolio. We therefore also tested {simaerep} under ideal conditions (200 patients, 20 sites, 0.5 AEs per visit, on average 20 visits per patient with SD 2, 1 under-reporting site with varying degrees of under-reporting).

Furthermore, we compared the tool's performance against two heuristic methods, a rank-based heuristic which always flags 5% (rounding up, sites with 0 AEs always get flagged) of the sites with the lowest AE reporting rates and one which uses boxplot statistics to detect lower boundary outliers of collected AE per visit rates.

We have also tested {simaerep} performance using non- default settings: using AE per days on study instead of AE per visit, using a deprecated method for determining the evaluation point visit_med75.

The primary performance metrics used for the comparison were the true positive rate (sensitivity) and false positive rate (1-specificity). All sites with an under-reporting probability greater than 95% were considered to be under-reporting.

The code and results are part of the {simaerep} documentation.

Boehringer-Ingelheim approach

Boehringer-Ingelheim evaluated {simaerep} under different conditions to understand the consistency and accuracy of the model performance and how early in a clinical trial AE under-reporting detection would be possible. The analyses conducted by the authors shed light on the method and suggest application of the {simaerep} model in practice for further enhancing its accuracy in assessing investigator site quality through remote evaluation of potential AE under-reporting.

Using 2 simulated clinical trial (CT) data sets of different sizes (n=7,000 subjects [sCT 1] and n=1,545 subjects [sCT 2]), the authors evaluated {simaerep}. While one department in Boehringer-Ingelheim built the data sets, the analyses were carried out by another department who remained blinded to the details about AE under-reporting built into the simulated data sets until all analyses were completed. This facilitated maintenance of the blinded state of the data analysis team and reduced the risk of bias in the results. AE rates were modeled using a Poisson distribution with region specific and trial risk population differences built in. In addition, trial initiation varied across regions and visits were modeled to randomly take place within a pre-specified time window. No missing visits were simulated. The characteristics of the data sets were modeled based on historic trials conducted by Boehringer-Ingelheim with subsequent introduction of under-reporting. Table B1 below shows additional characteristics of the simulated data sets.

Simulated data set characteristic	sCT1	sCT2
Number of Subjects	7000	1545
Number of Investigator Sites	490	172
Number of Subjects per Site	Minimum: 2	Minimum: 1
	Median: 13	Median: 7
	Maximum: 34	Maximum: 42
Number of CT visits per Subject (based on a fixed follow-up period per subject)	Baseline + 8 additional visits	Baseline + 8 additional visits
Number of Investigator Sites that are under-reporting AEs [% of all Sites]	80 [16.33%]	9 [5.23%]
Mean observed AE rate per 100 subject years	89.08	58.48
Rate of AE under-reporting across sites with under-reporting[1]	25% - 75%	25%

[1]The AE rate in a site simulated to under-report is derived as original AE rate multiplied by amount of AE under-reporting.

Table B1: Characteristics of Simulated Clinical Trials

Evaluations of the {simaerep} method were conducted on both data sets under different conditions. The analysis was performed in R version 4.1.2 using the {simaerep} R package version 0.4.0 The team first conducted a *Backward* evaluation which tested the ability of the model to detect AE under-reporting on a full data set, simulating the end of a clinical trial. The purpose of this evaluation was to test if the model works overall. A *Forward* evaluation was then conducted to test the ability of the model to detect AE under-reporting from data coming in periodically, simulating the model's performance in a clinical trial where data came in approximately monthly for about 2 years. The ability of the model to successfully detect AE under-reporting on a full data set (*Backward* evaluation) was thus a necessary precondition to test the model with data coming in periodically as would typically occur in a clinical trial (*Forward* evaluation).

For the *Backward* evaluation the team calculated the AUC of the ROC curve, the True Positive Rate (TPR), the False Positive Rate (FPR) as well as True Negative Rate (TNR) and False Negative Rate (FNR). The TPR defines the percentage of correctly classified AE under-reporting sites that are in fact AE under-reporting; the FNR defines the percentage of sites identified as non-under-reporting sites that are in fact AE under-reporting. For the *Forward*

evaluation, the team measured how early in the simulated clinical trials a site could first correctly be identified as AE under-reporting. In addition to looking for first signals, the team also evaluated how many days after the first subject visit it took for a site to be consistently identified as an AE under-reporter until trial end. This measure is called *Stable Detection*. Finally, because the {simaerep} model includes a correction for multiplicity the authors also evaluated the impact of the correction on the results to determine if its inclusion improved model performance.

Merck & Co., Inc., Rahway, NJ, USA approach

To demonstrate the efficacy of the method, the Merck & Co., Inc., Rahway, NJ, USA team performed testing of the algorithm using observations of protocol deviations (PD) related to AE under-reporting.

The following studies were included in the scope of the testing to cover the company's therapy areas of focus: General Medicine (Cardiovascular, Immunology, Neuroscience, Respiratory et.al), Infectious diseases and Vaccines (ID & Vac) (Antiviral, Antibacterial/Antifungal) and Oncology.

To obtain a sufficiently large sample for testing, we included three years of historical observations of AEs related to under-reporting of PDs. The algorithm was subsequently applied to studies in the sample, specifically those conducted on the date of occurrence of PDs related to AE under-reporting. We performed several experiments to assess the algorithm's performance, taking into account factors such as therapy area, number of study subjects, and number of visit_med75. Note: Visit_med75 is the median of the maximum visit of each patient at a site multiplied by 0.75.

Some observations of PDs were dropped from evaluation because the study at the date the PD occurred, did not have enough subjects (minimum number of 100 patients and distributed over at least ten sites) [Koneswarakantha, Björn, *et al.* "Follow-up on the use of advanced analytics for clinical quality assurance: bootstrap resampling to enhance detection of adverse event under-reporting." *Drug Safety* 44.1 (2021): 121-123)]. Under-reporting sites were the sites with a probability of under-reporting greater than 0.5 on the day the PD occurred.

We evaluated the performance of the algorithm using the rate of the number of sites that were predicted to have AE under-reporting and the number of the sites where the PD related to AE under-reporting were identified.

Results

Roche results

Our results demonstrate the effectiveness of the {simaerep} AE under-reporting detection tool in comparison with heuristic methods. Notably, {simaerep} significantly reduced the false-positive rate compared to both heuristics. However, it was observed that rank-based heuristics exhibited higher true positive rates, though this was accompanied by a consequential increase in false-positive rates Figure R1.

The boxplot outlier-based heuristic comes close to {simaerep} performance when it comes to both TPR and FPR but does not match it.

Our results also indicate that current default parameters for {simaerep} are optimized for most real-world scenarios. Under optimal conditions {simaerep} is capable of detecting nearly all under-reporting sites if the under-reporting rate exceeded 0.5, demonstrating its robustness.

When comparing the results to the boxplot outlier heuristic, the performance of {simaerep} was closest but with an overall better performance.

In conclusion, our results suggest that {simaerep} is a promising tool for AE under-reporting detection, offering superior performance to heuristic methods. Different AE under-reporting probability cut-offs could further be evaluated to increase TPR at the cost of also increasing FPR.

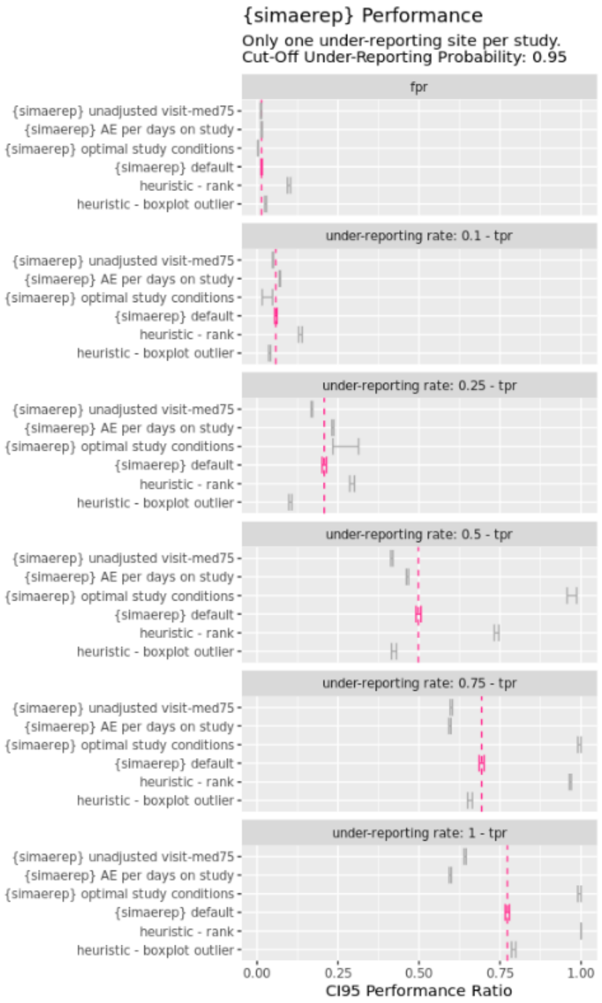

	fpr	
	ratio	CI95
{simaerep} unadj visit-med75	0.012	0.011 - 0.014
{simaerep} AE per days on study	0.014	0.013 - 0.016
{simaerep} optimal study conditions	0.002	0.001 - 0.004
{simaerep} default	0.014	0.012 - 0.016
Heuristic - rank	0.098	0.094 - 0.103
Heuristic - boxplot outlier	0.026	0.024 - 0.029
	Under-reporting rate: 0.1 - tpr	
	ratio	CI95
{simaerep} unadj visit-med75	0.049	0.048 - 0.050
{simaerep} AE per days on study	0.070	0.069 - 0.072
{simaerep} optimal study conditions	0.028	0.016 - 0.048
{simaerep} default	0.062	0.058 -0.065
Heuristic - rank	0.134	0.128 - 0.139
Heuristic - boxplot outlier	0.039	0.036 -0.042
	Under-reporting rate: 0.25 - tpr	
	ratio	CI95
{simaerep} unadj visit-med75	0.169	0.167 - 0.171
{simaerep} AE per days on study	0.234	0.231 - 0.237
{simaerep} optimal study conditions	0.272	0.234 - 0.314
{simaerep} default	0.213	0.206 - 0.219
Heuristic - rank	0.293	0.286 - 0.301
Heuristic - boxplot outlier	0.103	0.098 - 0.108
	Under-reporting rate: 0.5 - tpr	
	ratio	CI95
{simaerep} unadj visit-med75	0.416	0.413 - 0.419
{simaerep} AE per days on study	0.464	0.461 - 0.468
{simaerep} optimal study conditions	0.976	0.957 - 0.987
{simaerep} default	0.493	0.485 -0.501
Heuristic - rank	0.740	0.733 -0.746
Heuristic - boxplot outlier	0.423	0.415 - 0.431
	Under-reporting rate: 0.75 - tpr	
	ratio	CI95
{simaerep} unadj visit-med75	0.600	0.597 - 0.603
{simaerep} AE per days on study	0.595	0.592 - 0.599
{simaerep} optimal study conditions	1.000	0.990 - 1.000
{simaerep} default	0.695	0.688 - 0.702
Heuristic - rank	0.967	0.965 - 0.970
Heuristic - boxplot outlier	0.658	0.650 - 0.665
	Under-reporting rate: 1 - tpr	
	ratio	CI95
{simaerep} unadj visit-med75	0.642	0.639 - 0.645
{simaerep} AE per days on study	0.597	0.593 - 0.600
{simaerep} optimal study conditions	1.000	0.990 - 1.000
{simaerep} default	0.772	0.766 - 0.779
Heuristic - rank	1.000	1.000 - 1.000
Heuristic - boxplot outlier	0.792	0.786 - 0.799

Figure R1. {simaerep} performance in different simulated scenarios

{simaerep} default (pink): Performance with default parameters using simulated portfolio. *{simaerep} unadj visit-med75 /AE per days on study:* Performance with non-default parameters using simulated portfolio.
{simaerep} optimal study conditions: Performance under optimal conditions using homogenous simulated studies.
heuristic - rank: Performance of flagging the 5% of sites (rounding up) with the lowest AE per visit rate while always flagging sites with no AE using simulated portfolio.
heuristic - boxplot outlier: Performance of flagging lower-boundary outliers based on AE per visit boxplot statistics using simulated portfolio.
True positive rate (tpr), false positive rate (fpr), under-reporting probability theshold 95%

Boehringer-Ingelheim results

Backward evaluation results are shown below in Table B2. The team evaluated the AUC of the ROC curve as part of the *Backward* analysis. The model with the correction for multiplicity showed a bigger difference in the AUC results. The overall AUCs are 0.86 (sCT2) and 0.92 (sCT1) for the model with correction and 0.93 (sCT1) and 0.94 (sCT2) for the model without the correction. In addition, the model with the correction for multiplicity showed a bigger difference of TPR results between the 2 different simulated patient data sets (sCT1: 91.25% vs sCT2: 22.22%) than the model without the correction for multiplicity (sCT1: 87.50% vs sCT2: 88.89%). The same was true of the FNR for the model with the correction (sCT1: 8.75% vs sCT2: 77.78%) as compared to results of the model without the correction (sCT1: 12.50% vs sCT2: 11.11%).

	Clinical Trial Data Set	AUC of ROC	TPR[1]	FNR[1]	TNR[1]	FPR[1]
Model *with* correction for multiplicity	sCT1	0.9191	91.25%	8.75%	75.61%	24.39%
	sCT2	0.8634	22.22%	77.78%	99.39%	00.61%
Model *without* correction for multiplicity	sCT1	0.9317	87.50%	12.50%	85.12%	14.88%
	sCT2	0.9377	88.89%	11.11%	59.51%	40.49%

[1]Respective threshold from optimal Youden J used.

Table B2: Results of Backward Test on sCT1 and sCT2 Data Sets, with and without the Correction for multiplicity

The results of the *Forward* evaluation on the sCT1 data set are presented below in Figures B1 and B2. Each line represents one true AE under-reporting site, the blue dots represent the time point at which a site has been classified as potential AE under-reporter for the first time. The horizontal axis indicates the time in days from first subject visit onwards. The vertical axis is a proxy of available data and depicts the cumulative number of subject visits at site. Without the

correction, the first signal of AE under-reporting for the sCT1 appears at 35 days whereas with the correction, it appears at 163 days. A similar pattern of results was observed on the sCT2 data set as shown in Figures B3 and B4.

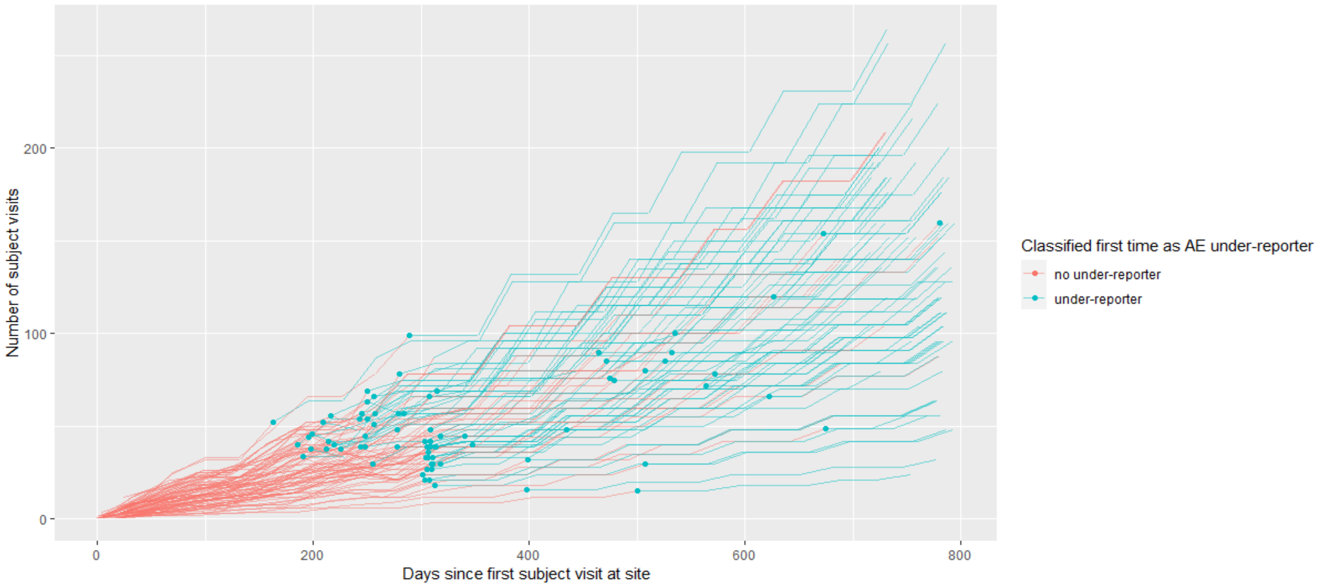

Figure B1 - Forward Test with correction for sCT1, subset of all true AE under-reporting sites

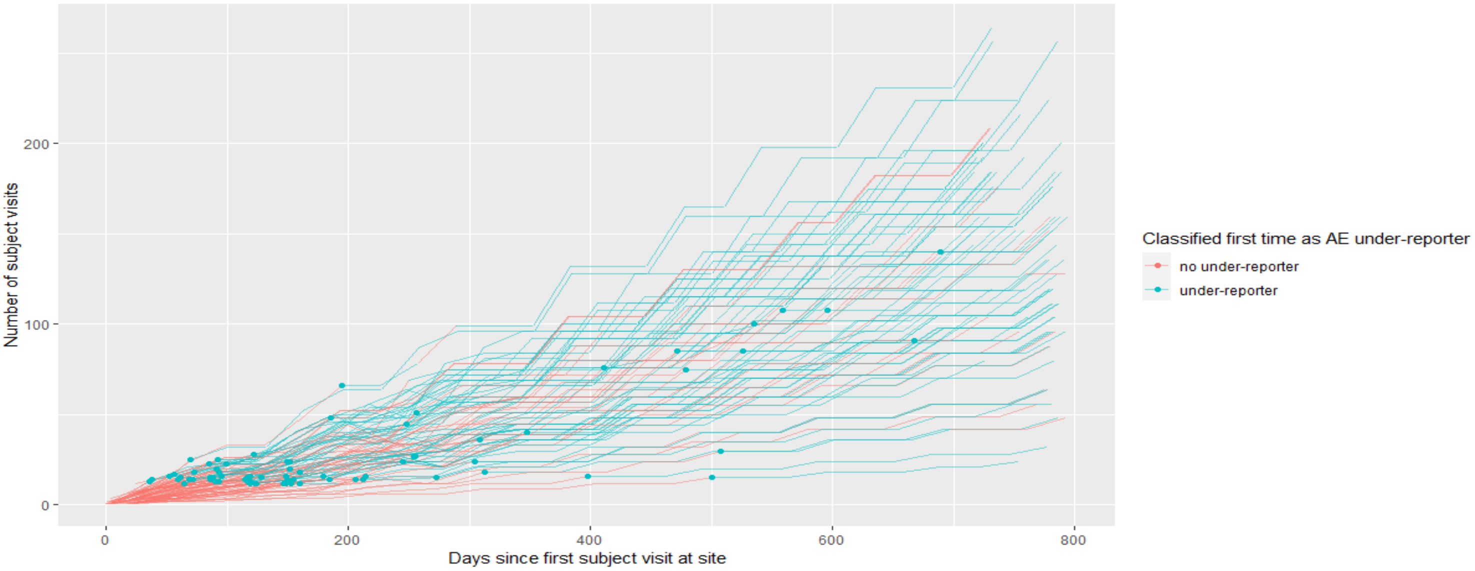

Figure B2: Forward Test without correction for sCT1, subset of all true AE under-reporting sites

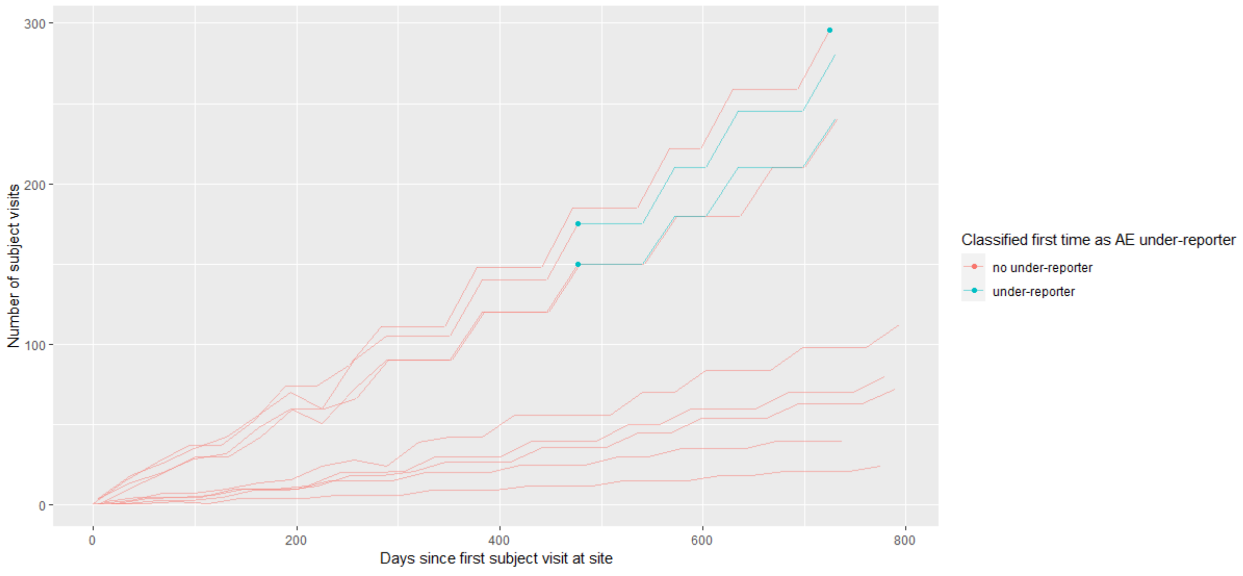

Figure B3: Forward Test with correction for sCT2, subset of all true AE under-reporting sites

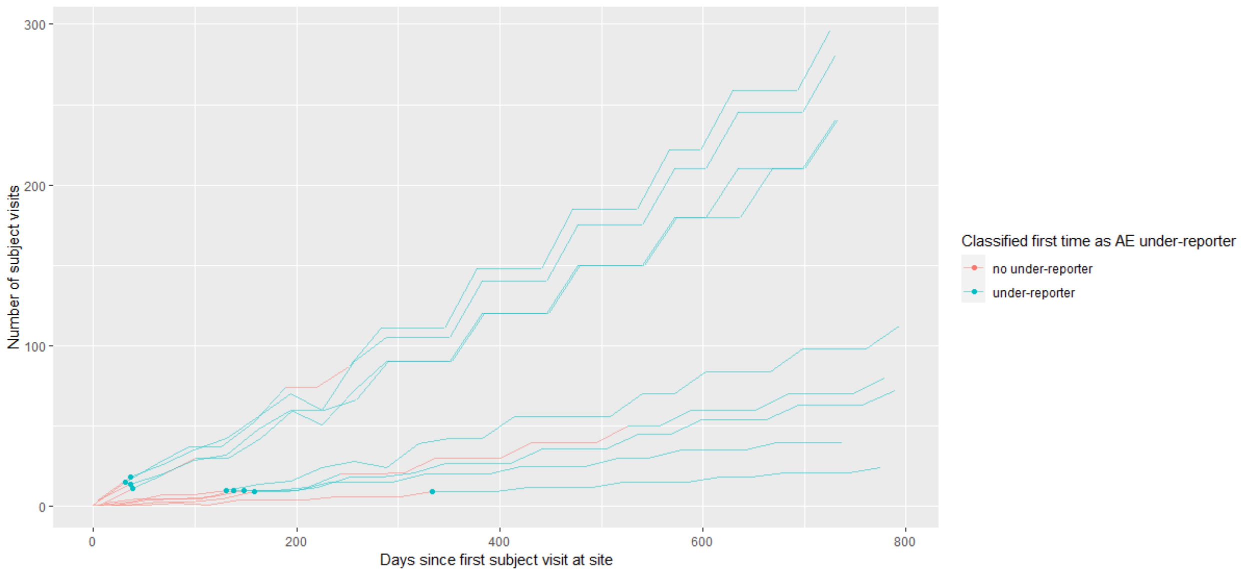

Figure B4: Forward Test without correction for sCT2, subset of all true AE under-reporting sites

Table B3 below presents the number of days from first subject clinical trial initiation visit until *Stable Detection* (the number of days after the first subject visit it took for a site to be consistently identified as an AE under-reporter until trial end) of AE under-reporting according to 4 different detection proportions levels: 10%, 50%, 75% and 95%. It is noteworthy that the model without the correction showed *Stable Detection* among 10% of correctly identified true AE under-reporting sites as early as 36 days (sCT2) and 68 days (sCT1) following the first subject visit. For the sCT1 data set, the total number of correctly identified sites was nearly the same (with correction: 73 out of 80 sites; without correction: 70 out of 80 sites) at the end of the simulated trial, however the number of days until 10% of the sites have been identified with stable detection differs (219 days with correction vs. 68 days without correction). The difference in the time to *Stable Detection* between the model with and without the correction for multiplicity begins to narrow until only a 15-day difference (with correction n = 674 days vs without correction n = 689 days) at the 95% quantile. The results for time to *Stable Detection* for the sCT2 are substantially different between the model with and without correction. As shown in Table B3 below, the *Forward* evaluation, the model with the correction performed worse than the model without the correction: With the correction n = 2 sites identified; without the correction n = 8 sites identified.

	Clinical Trial Data Set	Proportion of correctly classified AE under-reporting sites				Total number of sites correctly classified at trial end [N]
		10%	50%	75%	95%	
		Time since first subject visit until stable detection [days]				
Model *with* correction for multiplicity	sCT1	219	308	473	674	73
	sCT2	477	477	725	725	2
Model *without* correction for multiplicity	sCT1	68	219	398	689	70
	sCT2	36	131	158	526	8

Description of results for first line: The proportion of 10% of correctly classified sites is 7 out of the 73, while 73 sites were correctly classified as AE under-reporting sites at trial end and present 91.25% of the sites that are actual AE underreporters as per Table B2.

Table B3: Days since first subject visit until X% of correctly classified AE-under-reporting sites (true positive) have "Stable Detection", with and without the Correction for multiplicity

Merck & Co., Inc., Rahway, NJ, USA results

The dataset for the analysis includes the last 3 years of historical data of studies for which PDs related to AE under-reporting were detected. Four experiments were performed on this dataset to evaluate the performance of the algorithm. The experiments are replicated for every therapy area. In the first experiment, all studies that had PDs related to AE under-reporting for which the algorithm was applicable were considered.

For the next two experiments, we limited to the studies that have more than 5 or 10 subjects randomized on site.Subset of studies for which the sites had visit_med75 at least 10 were considered for the third and fourth experiment. The results of the four experiments are presented in Table M1.

Performance(%) (cutoff=0.5)	All data	No of patients on site >=5	No of patients on site >=10	No of patients on site >=5 & Visit_med75 >=10	No of patients on site >=10 & Visit_med75 >=10
**All therapy areas**	20.05%	25.6%	32.82%	45.9%	71.42%
**Oncology**	10.92%	14.09%	18.03%	20.58%	36.4%
**Vaccines**	39.84%	49.41%	55.17%	80.77%	88.23%
**General Medicine**	5.5%	6.25%	-	-	-

Table M1: Performance of the algorithm with different filters on the data.

The findings indicate that the algorithm's effectiveness improves as the number of participants and visits increases. Furthermore, Table M1 illustrates that the algorithm performs better in identifying under-reporting sites in vaccine studies than in other therapy areas. This could be attributed to the significantly higher number of patients enrolled in ID & Vac, as demonstrated in Figure M1 (p-value<0.05 based on negative binomial regression model), compared to Oncology.

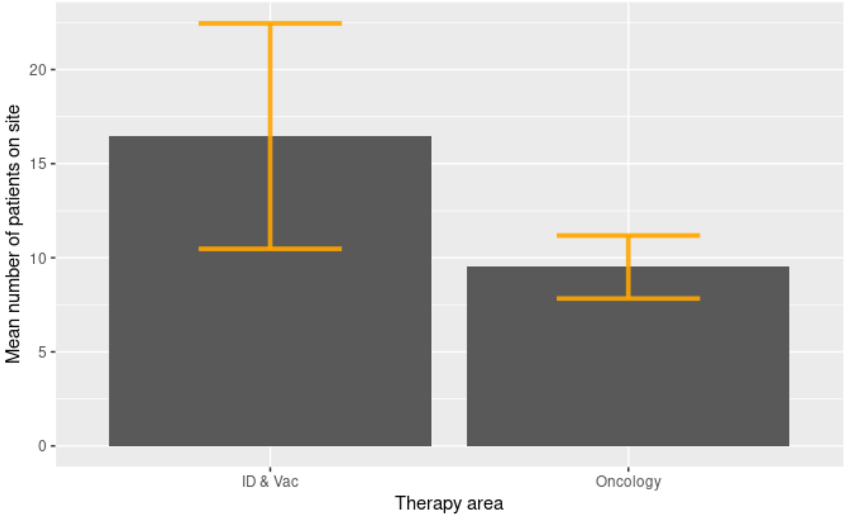

Figure M1 - Number of patients on site for studies from ID & Vac and Oncology

Additionally, we delved into why the algorithm was unable to identify sites that were genuinely under-reporting. To this end, we examined the relationship between the average adverse events (AE) reported by the site and the average AE reported by the study at visit_med75. We discovered that in 30% of the under-reporting cases, the average AE reported by the site at visit_med75 was higher than the site-level average AE reported by the study. These instances cannot be detected by the algorithm because it only identifies sites with a (significantly) lower average than the study. The algorithm's primary function is to pinpoint under-reporting on sites with a markedly lower average of AEs when compared to the overall study at visit_med75.
